# Acinic cell carcinoma of the breast: A comprehensive review

**DOI:** 10.1016/j.breast.2022.10.012

**Published:** 2022-10-28

**Authors:** Azra Ajkunic, Faruk Skenderi, Nada Shaker, Saghir Akhtar, Janez Lamovec, Zoran Gatalica, Semir Vranic

**Affiliations:** a Fred Hutchinson Cancer Research Center, Seattle, WA, USA; b Sarajevo School of Science and Technology, Sarajevo, Bosnia and Herzegovina; c Department of Pathology, The Ohio State University Wexner Medical Center, Columbus, OH, USA; d College of Medicine, QU Health, Qatar University, Doha, Qatar; e Institute of Oncology Ljubljana, Ljubljana, Slovenia; f Department of Pathology, University of Oklahoma Health Sciences Center, Oklahoma City, OK, USA

**Keywords:** Breast cancer, Special types, Salivary gland-type tumors, Acinic cell carcinoma

## Abstract

Acinic cell carcinoma of the breast is a rare special subtype of breast cancer in the category of salivary gland-type tumors. It is morphologically similar to acinic cell carcinomas of salivary glands and pancreas and has a triple-negative phenotype (estrogen receptor-negative, progesterone receptor-negative, and Her-2/neu negative). Its molecular genomic features are more similar to triple-negative breast cancer of no special type than to its salivary gland counterpart. However, the clinical course of the mammary acinic cell carcinoma appears to be less aggressive than the usual triple-negative breast carcinomas. This review comprehensively summarizes the current literature on the clinicopathologic, immunohistochemical, and molecular features of this rare and distinct subtype of breast cancer.

## Highlights

• Acinic cell carcinoma of the breast is a very rare subtype of breast cancer.
• It is morphologically similar to acinic cell carcinomas of salivary glands and pancreas and has a triple-negative phenotype.
• Its molecular genomic features are more similar to TNBC NST than to its salivary gland counterpart.
• The clinical course of the mammary acinic cell carcinoma appears to be less aggressive than the usual TNBC.

## 1 Introduction

Breast cancer is the most common malignancy in women worldwide and the second deadliest after lung and bronchus primary [1]. Nevertheless, it is a heterogeneous and complex disease at the morphologic, molecular genetic, and clinical levels [2].

Invasive ductal carcinoma no special type (NST) is the most common subtype of breast cancer and accounts for >70% of all breast cancers [3]. The remaining 30% comprises so-called special types of breast cancer, represented in >15 morphologic and molecular subtypes.

**Fig. 1:**
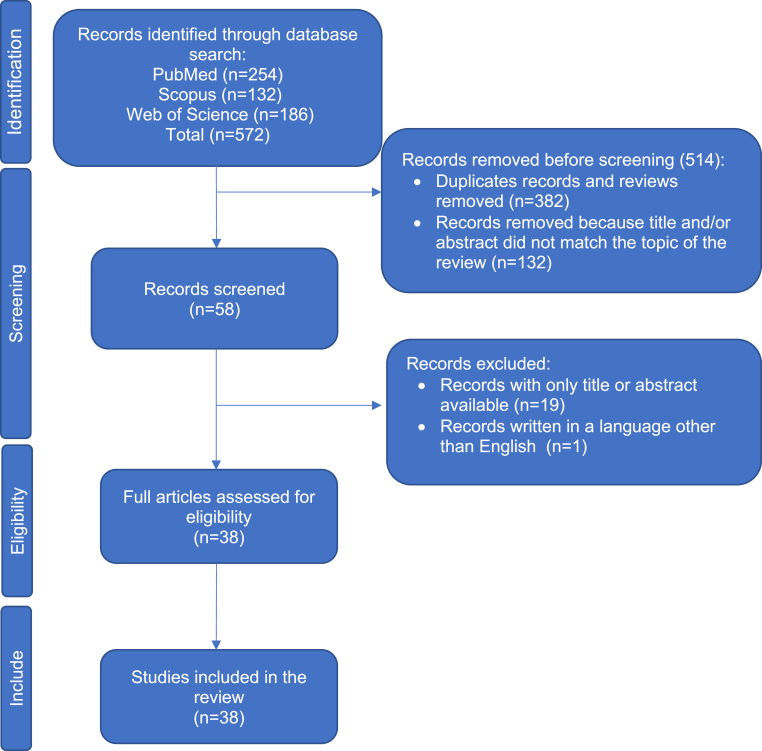
A flowchart shows the literature search approach for identifying and analyzing acinic cell carcinoma studies.

A rare subgroup of the special type of breast cancer is salivary gland-like carcinomas. This review comprehensively summarizes the current knowledge on the specific variant of salivary gland-like carcinoma of the breast, known as acinic cell carcinoma (AcCC). Our literature search included PubMed/MEDLINE, Scopus, and Web of Science (Science Citation Index/Science Citation Index Expanded). The most recent literature search was performed in May 2022 (see flowchart in Fig. 1).

### 1.1 Salivary gland-type tumors of the breast

Both salivary glands and breasts are composed of tubuloacinar glands with similar histologic structures. Acinar cells, which constitute the major component of salivary glands, have been found in breast lobules, and various types of neoplasms may occur indiscriminately in both tissues. Tumors that develop in the mammary and salivary glands share morphologic and immunohistochemical features. However, their incidence and clinical behavior differ substantially [3]. The defining genomic abnormalities in certain types of primary salivary glands’ tumors may also be detectable in the salivary gland-type primary tumors of the breast (e.g., adenoid cystic carcinoma with t(6; 9), leading to the *MYB-NFIB* fusion, secretory carcinoma with t(12; 15) and the *ETV6-NTRK3* fusion, or pleomorphic adenoma with *HMGA2* or *PLAG1* rearrangements) [4].

Salivary gland tumors of the breast encompass a rare and diverse group of neoplasms, including adenoid cystic carcinoma, secretory carcinoma, mucoepidermoid carcinoma, polymorphous adenocarcinoma, and acinic cell carcinoma [3,5]. A distinct group of salivary gland tumors of the breast includes epithelial-myoepithelial tumors, some of which are benign (pleomorphic adenoma and adenomyoepithelioma), while adenoid cystic carcinoma and malignant adenomyoepithelioma are malignant [3].

AcCC is a rare malignant tumor, morphologically and ultrastructurally comparable to acinic cell carcinoma of the parotid gland. Roncaroli et al. were the first to describe this form of breast cancer in 1996 [6]. Although most breast cancers are ductal and show neither acinar nor secretory differentiation, invasive ductal carcinomas may rarely show lysosome-positive cells with granular cytoplasm. Secretory carcinoma is typically attributed to breast cancers associated with a pathognomonic *ETV6-NTRK3* gene fusion. However, a broader subclass of breast carcinomas includes AcCC and cystic hypersecretory carcinomas that mimic the prosecretory phenotype of a lactating breast [7].

### 1.2 Epidemiology and clinical features of mammary AcCC

The actual incidence of AcCC is unknown; as of 2018/2019, approximately 50 cases have been published in the literature [3]. The largest study based on a database search included 11 patients from China [8]. A study by Zhang and colleagues (2020) identified 582 cases of triple-negative breast carcinoma (TNBC), with only one case described as AcCC (frequency of 0.2%) [9]. Our comprehensive literature search identified 68 cases published in the English-language literature between 1996 and 2022 (summarized in Table 1). We comprehensively searched PubMed/MEDLINE, Scopus, and Web of Science Core Collection (Science Citation Index Expanded/SCIE/) using the following keywords: “acinic cell carcinoma”, “breast”, “breast carcinoma with acinic cell differentiation”, “breast carcinoma and microglandular adenosis”. Conference proceedings (e.g., European Congress of Pathology, published in Virchow Archives, and Annual Meeting of the United States and Canadian Academy of Pathology/USCAP/, published in Modern Pathology/Laboratory Investigation) were excluded. These references did not include full abstracts in SCIE (Fig. 1). Non-English language references were also excluded (Fig. 1).

**Table 1:** A summary of clinical and histopathologic characteristics of the acinic cell carcinoma of the breast. The studies were identified through a comprehensive literature search in the major databases (PubMed/MEDLINE, Scopus, and Web of Science Core Collection).

Author (year)	Sex/Age	Site	Tumor size (mm)	Growth pattern	Radiographic features	Node status	Follow up (months)	Recurrences/Metastases/Outcome
1 Sarsiat et al. (2022) [10]	F/59	R	71	Solid and microglandular	Mammography: An ill‐defined solid lesion.	0/2	49	Peritoneal metastases/DOD
2 Yu et al. 3 (2022) [11]	F/49 F/42	L uiq R uoq	35 30	Solid and microglandular Solid, microglandular and pseudolobular	Mammography: Occult cancer. Mammography: Detected during screening	0/3 0/3		
4 Waever et al. (2021) [12]	F/42	L	60	Solid, microlobular and pseudolobular	Ultrasonography: Well-defined, lobulated, solid lesion with characteristics of a fibroadenoma		48	NED
5 Sen et al. (2018) [13]	F/41	R uoq	25	Acinar and solid	Ultrasonography and mammography: No abnormalities.	0	12	NED
6 Li et al. (2017) [14]	F/52	L luq	15	Cystic and cribriform		0/1	3	AW
7 Kim et al. 8 (2017) [15] 9	F/38 F/47 F/60	L L L				0 1a 0		NED NED NED
10 Kawai et al. (2016) [16]	F/49	R	35	Solid-trabecular and acinar	Mammography: Lobulated mass with poorly defined margins, without microcalcifications Ultrasonography: A hypoechoic mass with irregular margins.		8	DR
11 Xu et al. (2016) [17]	F/41	R uoq	12	Acinar-glandular and solid	Mammography: A small oval radiopaque mass		13	
12 Sherwell- Cabello et al. (2016) [18]	F/38			Microglandular				NED
13 Conlon et al. 14 (2016) [7]	F/47 F/49	R luq R uoq	23 11	Microglandular and solid Microglandular and solid	Mammography: Occult Ultrasonography: Solid mass associated with innumerable anechoic cysts	1/18 0	72 18	DR/Alive with disease NED
15 Guerini-16 Rocco et 17 al. (2015) [19] 18 19 20 21 22	F/36 F/55 F/34 F/42 F/34 F/48 F/70 F/35		50 19 11 36 20 14 18	Clear cell Microglandular Microglandular Microglandular Microglandular Microglandular Microglandular Microglandular		10/17 0/8 0/3 2/22	24 132 15 60 72	DOD NOD NED NED DR DR
23 Piscuoglio 24 et al. (2015) [20]	F/49 F/45		15 21	Microglandular Microglandular				
25 Zhong et al. 26 (2014) [8] 27 28 29 30 31 32 33 34 35	F/50 F/40 F/59 F/42 F/56 F/42 F/50 F/61 F/35 F/34 F/46		15 16 30 17 30 28 50 15 18 25 30			0 0 0 0 0 0 0 0 0 0 0	22 17 10 10 24 15 40 22 64 60 10	NED NED NED NED Lung metastasis NED NED NED NED NED NED
36 Limite et al. (2014) [21]	F/26	R lqu	16	Acinic-glandular and solid		0/1	8	
37 Falleti et al. (2013) [22]	F/58	R peri-areolar	30	Solid, microglandular and microacinar	Mammography: Thickening with microcalcifications, firm, nodular lesion.	0/1	10	AW
38 Osako et al. 39 (2013) [23] 40	F/50 F/37 F/46		52 22 50	Microglandular Microglandular and solid Papillary, microglandular, solid and cystic		0/30 0/23 0/4	184.8 139.2 64.8	NED NED NED
41 Shingu et al. (2013) [24]	F/41	L loq	35	Solid, trabecular and microglandular	Mammography: Focal asymmetric density of the breast. Ultrasonography: A heterogeneous hypoechoic mass with ill-defined margins. MRI: High-intensity mass, but no intraductal dissemination.	0/1	36	NED
42 Winkler et al. (2013) [25]	F/56	R	47	Solid	MRI: Heterogeneous segmental non-mass-like enhancement extending toward the nipple in a triangular configuration.	0/1	24	NED
43 Zhao et al. (2014) [26]	F/38	R uoq	30	Microglandular		0/23	10	NED
44 Ripamonti et al. (2013) [27]	F/44	L uoq	13	Microglandular	MRI: Solid mass with pushing borders.	0/2	19	AW
45 Choh et al. (2012) [28]	F/79	L upper pole	27			0/1	9	NED
46 Sakuma et al. (2013) [29]	F/61	R uoq	14	Solid and microcystic	Mammography: Dense, inhomogeneous parenchyma; Ultrasonography: A hypoechoic, irregularly shaped mass with ill-defined borders.	0	14	NED
47 Chang et al. (2011) [30]	F/39		55	Acinar/glandular and solid	Ultrasonography and mammography: No abnormalities.	1		Unfavorable prognosis
48 Huo et al. (2011) [31] 49 50	F/40 F/30 F/51	R uoq R L	36 26 21	Microglandular, solid and focally microcystic Microglandular Microglandular		1/21 2/33 0	12 34 26	NED Bone metastasis/DOD NED
51 Stolnicu et al. (2010) [32]	F/79	L uoq	25	Solid, microcystic, microglandular and trabecular	Ultrasonography and mammography: Malignant features (no details provided).	10/15	9	AW
52 Matoso et al. (2009) [33]	F/62	L		Amphophilic cytoplasm with zymogen-like secretory granules				
53 Tanahashi et al. (2007) [34]	F/80	R uoq	21	Acinar	Ultrasonography: A hypoechoic nodule with a smooth surface. Mammography: A well-demarcated mass. No microcalcifications.	0/1	22	AW
54 Peintinger et al. (2004) [35]	F/36	R	35	Solid and microglandular		0/15	120	Lung metastasis/NED 12 months after surgery
55 Kahn et al. (2003) [36]	F/56	L	22	Solid		0/18	28	NED
56 Hirokawa et al. (2002) [37] 57 58	F/20 F/61 F/59	R L R	27 25 10	Papillary/cystic Papillary/cystic, microcystic and follicular Microfollicular	Mammography and ultrasonography: A solid lesion. Mammography and ultrasonography: An irregular-shaped mass with microcalcifications.	0 0 0	6 24 94	AW NED NED
59 Coyne et al. (2002) [38]	F/49	R	20	Microglandular		2/11	36	Liver metastasis/DOD
60 Damiani et 61 al. (2000) [39] 62 63 64 65	F/42 F/35 F/63 F/55 F/64 F/80	R uoq R uoq L L L uiq R uoq	30 40 50 20 33 20	Solid Microglandular Microglandular Microglandular Microglandular Microglandular		1/18 2/20 0/8	60 12 48 Lost 12 12	AW AW DR AW AW
66 Schmitt et al. (2000) [40]	F/79	L uiq	45	Solid	Mammography: A round and well-circumscribed lesion.	0/23	21	NED
67 Shimao et al. (1998) [41]	M/23	L	48	Solid	Ultrasonography: A clear marginal cystic mass composed of hypoechoic intracystic fluid and a hyperechoic intracystic tumor		34	NED
68 Roncaroli et al. (1996) [6]	F/42	R uoq	30	Solid	Mammography: A mass with well-defined margins with scattered granular calcifications.	1/18	60	AW

F – female; M − male; AW-Alive and well; DOD-Died of disease, DR-disease recurrence, ioq-inferior outer quadrant; L-left; loq-lower outer quadrant; lqu-lower quadrant union; R-right; NED-no evidence of disease, uiq-upper internal quadrant; uoq-upper outer quadrant.

AcCC of the breast affected women between 20 and 80 years (mean 48.5 years, median: 47 years). Only a single male case has been reported [41]. As with other breast cancer subtypes, there was no predilection for any particular site in AcCC. The clinical and radiologic appearances are similar to that of other breast cancer subtypes, e.g., as a palpable, poorly definable lump/mass with microcalcifications; occasionally, other clinical and radiologic appearances may occur (e.g., lesion without a mass, radiologically hidden cancer, or small oval radiopaque mass) (Table 1). The tumor size of AcCC varies from 10 mm to 71 mm, but two independent case series reported that the median size was 19 and 25 mm, respectively [20,26]. Axillary lymph node metastasis appears to be a rare event in AcCC (Table 1). The largest series by Zhong et al. (2014) reported no axillary metastases in all eleven included patients [8]. However, local recurrence was reported in several independent case series of AcCC (Table 1). The pattern of distant metastases is similar to that of invasive ductal carcinoma NST, involving lung, liver, and bone [3,42] and is usually associated with an unfavorable clinical outcome (Table 1). Occasionally, peritoneal metastases may also develop [10] but other common (brain) and unusual visceral metastases (e.g., genitourinary, reproductive tract) have not been reported yet for patients with AcCC.

The therapeutic approach for patients with AcCC has been similar to patients with invasive ductal carcinoma NST (Table 2). The treatment modalities follow the general breast cancer guidelines and include surgery with axillary lymph node dissection (some with sentinel lymph node biopsy); Adjuvant chemo- and radiotherapy were also used, as well as hormonal therapy for the hormone-receptor-positive cases [[25], [27], [31], [39]]. Several patients were also treated with neoadjuvant chemotherapy (Table 2) [7,10,25,[12], [17], [31], [38], [39]]. Given a relatively indolent clinical course in a subset of AcCC (e.g., low-grade cases), these patients should probably be spared chemotherapy [43]; similarly, neoadjuvant chemotherapy should not be a preferable option for AcCC patients due to the predominantly low proliferation rate measured by Ki-67 (Table 3).

**Table 2:** Overview of the reported treatment modalities used for the treatment of acinic cell carcinoma of the breast.

Author (year)	Surgery	Adjuvant therapy
1 Sarsiat et al. (2022) [10]	BCS + SLND	Neo-CT
2 Yu et al. 3 (2022) [11]	MRM + SLND BCS + SLND	– –
4 Waever et al. (2021) [12]	Mastectomy + SLND	Neo-CT
5 Sen et al. (2018) [13]	MRM + ALND	–
6 Li et al. (2017) [14]	Mastectomy	–
7 Kim et al. 8 (2017) [15] 9	BCS BCS BCS	CT + RT CT + RT –
10 Kawai et al. (2016) [16]	MRM + SLND	CT
11 Xu et al. (2016) [17]	BCS	Neo-CT
12 Sherwell-Cabello et al. (2016) [18]	na	na
13 Conlon et al. 14 (2016) [7]	B/L mastectomy + ALND BCS	CT Neo-CT
15 Guerini-16 Rocco et 17 al. (2015) [19] 18 19 20 21 22	na na na na na na na na	na na na na na na na na
23 Piscuoglio 24 et al. (2015) [20]	na na	na na
25 Zhong et al. 26 (2014) [8] 27 28 29 30 31 32 33 34 35	BCS BCS Mastectomy BCS Mastectomy Mastectomy Mastectomy Mastectomy BCS Mastectomy Mastectomy	CT + RT CT + RT CT CT + RT CT CT CT CT CT + RT CT CT
36 Limite et al. (2014) [21]	BCS + SLND	–
37 Falleti et al. (2013) [22]	BCS + SLND	–
38 Osako et al. 39 (2013) [23] 40	MRM + ALND BCS + ALND MRM + SLND	– – –
41 Shingu et al. (2013) [24]	BCS + SLND	CT + RT
42 Winkler et al. (2013) [25]	MRM + SLND	Neo-CT + HT
43 Zhao et al. (2014) [26]	MRM + ALND	CT
44 Ripamonti et al. (2013) [27]	MRM + SLND	HT
45 Choh et al. (2012) [28]	BCS + SLND	RT
46 Sakuma et al. (2013) [29]	BCS + ALND	–
47 Chang et al. (2011) [30]	BCS + ALND	–
48 Huo et al. 49 (2011) [31] 50	MRM + ALND BCS + ALND BCS + SLND	Neo-CT + RT + HT CT + RT
51 Stolnicu et al. (2010) [32]	MRM + ALND	–
52 Matoso et al. (2009) [33]	na	na
53 Tanahashi et al. (2007) [34]	MRM + SLND	–
54 Peintinger et al. (2004) [35]	BCS + ALND	CT + RT
55 Kahn et al. (2003) [36]	MRM + ALND	–
56 Hirokawa et 57 al. (2002) [37] 58	MRM + ALND MRM + ALND MRM + ALND	– – –
59 Coyne et al. (2002) [38]	MRM + ALND	Neo-CT + CT
60 Damiani et 61 al. (2000) [39] 62 63 64 65	MRM MRM + ALND BCS BCS BCS + ALND BCS	CT Neo-CT -- – HT
66 Schmitt et al. (2000) [40]	MRM + ALND	RT
67 Shimao et al. (1998) [41]	BCS + ALND	–
68 Roncaroli et al. (1996) [6]	MRM + ALND	CT

ALND – Axillary lymph node dissection; BCS – Breast-conserving surgery; B/L – Bilateral; CT – Chemotherapy (adjuvant); HT – Hormone therapy; MRM – Modified radical mastectomy; Neo-CT – Neoadjuvant chemotherapy; RT – Radiation therapy; na – Not available; SLND – Sentinel lymph node dissection.

**Table 3:** Summary of the immunohistochemical features reported in the literature on mammary acinic cell carcinoma.

Immunohistochemical biomarker	Positivity, % (number of cases/total cases)
*Steroid receptors*	
Estrogen receptor	10 (7/66)
Progesterone receptor	11 (7/62)
Androgen receptor	10 (1/10)
*Growth factor receptors*	
HER-2/neu	0 (0/54)
EGFR	67 (2/3)
*Cytokeratins*	
panCK	100 (11/11)
LMW-CK	100 (1/1)
HMW-CK	0 (0/1)
CK5/6	50 (3/6)
CK7	100 (9/9)
CK14	0 (0/1)
CK18	100 (1/1)
CK20	0 (0/2)
*Other biomarkers*	
Ki-67	Range: 5–71% (most studies: 5–30%)
E-cadherin	100 (7/7)
Beta-catenin	100 (1/1)
Lysozyme	95 (39/41)
Amylase	94 (17/18)
_α1-_ACT	96 (25/26)
GATA3	50 (2/4)
GCDFP-15	56 (14/25)
S-100	93 (50/54)
EMA	100 (29/29)
SMA	0 (0/14)

α1-ACT - α1 anti-chymotrypsin/trypsin; CK – Cytokeratin; EGFR – Epidermal growth factor receptor; EMA – Epithelial membrane antigen; GDCDFP-15 – Gross cystic disease fluid protein; HER2 – Human epidermal growth factor receptor; HMW – High molecular weight; LMW – Low molecular weight; SMA – Smooth muscle actin.

### 1.3 Histopathology of AcCC

**Fig. 2:**
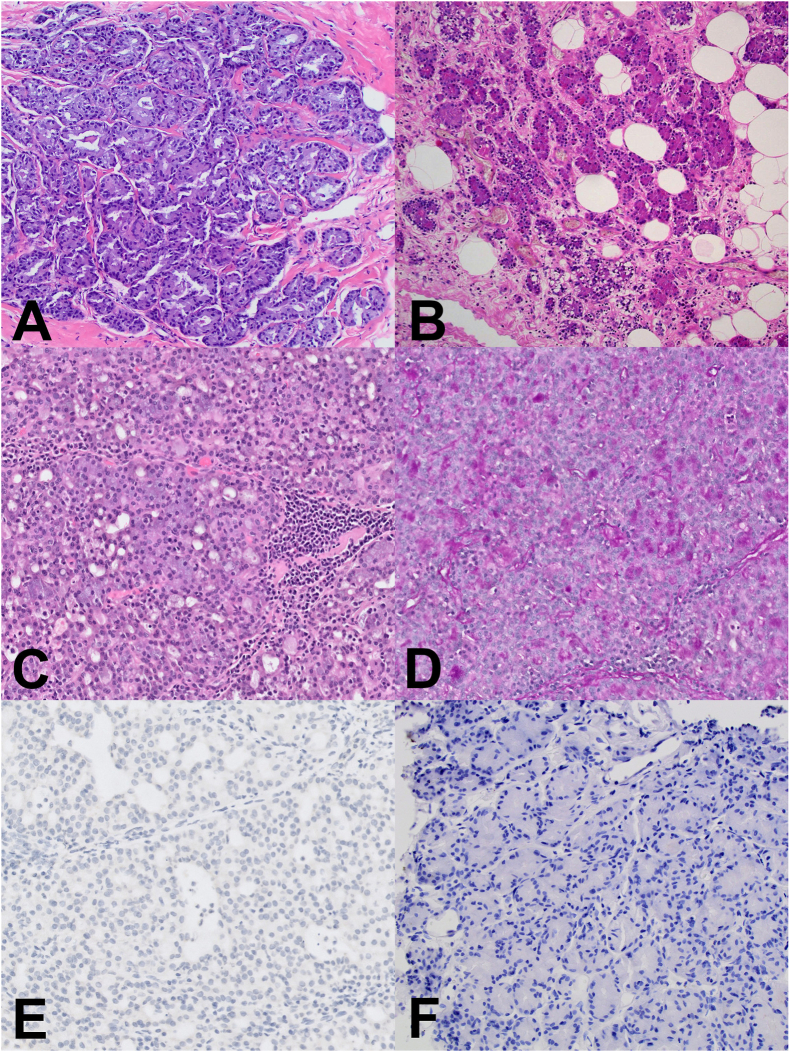
A-F Hematoxylin and Eosin (H&E) slides of a case of acinic cell carcinoma of the breast with different growth patterns, including acinar (A), microglandular (B), and solid (C) growth patterns. Image D shows PAS positivity in tumor cells, while estrogen receptor (E), and GATA3 (F) are negative [47].

According to the current World Health Organization classification (WHO) from 2019 [3], AcCC is defined as TNBC and is recognized as a distinct form of salivary gland-type tumor of the breast (Table 3, Table 4). Morphologically, AcCC consists of serous differentiation cells containing zymogenic granules in the cytoplasm. These granules stain positive for Periodic Acid-Schiff (PAS) with diastase (PAS-D) (Fig. 2D). It exhibits various growth patterns, with cells arranged in solid and/or microglandular growth patterns (Fig. 2A–C) (various growth patterns and morphological features of AcCC are summarized in Table 1). Therefore, recognition of the cytologic features is critical for proper diagnosis. The neoplastic cells have abundant eosinophilic or basophilic granular cytoplasm with centrally located nuclei and prominent nucleoli [3] (Fig. 2). The neoplastic cells may occasionally have a clear cytoplasm. Atypia is usually more prominent in solid areas. Mitotic figures may also be seen but are usually not marked. AcCC may be accompanied by invasive ductal carcinoma NST (“mixed” cases), as documented in some studies [20,19]. In rare cases, AcCC may be associated with metaplastic breast carcinoma [19] or other salivary gland-type tumors such as high-grade/basaloid/forms of adenoid cystic carcinoma. An in situ component may also be present within the AcCC mass, indicating the primary mammary origin of the neoplasm (usually high-grade ductal carcinoma in situ/DCIS/) [3].

**Table 4:** Overview of the studies exploring molecular genetic characteristics of acinic cell carcinoma of the breast.

Author (year)	Cases	Method(s)	Steroid receptors and HER-2/neu status	Molecular features (frequency)
Waever et al. (2021) [12]	1	IHC, NGS	Triple-negative	DOG1 positive, GATA3 positive *TP53, RET* SNV mutation
Beca et al. (2019) [44]	3	WES, RNA sequencing	Triple-negative (3/3)	*TC2N-FBLN5* (2/3), *TP53* (2/3), *MLH1* (1/3*), CTNNB1* (1/3), a *BRCA1* homozygous deletion (1/3), high-level amplification of 12q14.3–12q21.1 in *MDM2, HMGA2, WIF1*, *FRS2, PTPRB* (1/3), focal amplification in 20p12.3 encompassing *PCNA* (1/3)
Geyer et al. (2017) [45] a	8	IHC, massively parallel sequencing	Triple-negative (8/8)	Lysozyme (8/8*)*^a^ *TP53* (7/8)^a^, *ERBB4* (2/8)^a^, *ERBB3* (1/8)^a^, *BRCA1* (1/8)^a^, *FGFR2* (1/8)^a^, *PIK3CA* (1/8)^a^, *INPP4B* (1/8)^a^ Complex and multiple genomic alterations: Gains of 1q, 2q, 7p, and 8q and losses of 3p, 5q, 6q, 14q, 17p and 17q
Conlon et al. (2016) [7]	2	IHC, NGS	ER(−), PR (+), HER2 (−) (I); ER (−), PR (−), HER2 (−) (II)	α1-ACT (2/2), lysozyme (2/2), GCDFP-15 (1/2) TP53 (1/2), MLL3 (1/2), TSC2 (1/2)
Guerini-Rocco et al. (2015) [19],^a^	8	IHC, massively parallel sequencing	Triple-negative (8/8)	Lysozyme (8/8), Ki-67 (11–27%) *TP53* (7/8), *KMT2D* (2/8), *NEB* (2/8), *EPPK1* (2/8), *CUBN* (1/8) *INPP4B* (1/8), *FGFR2* (1/8), *PIK3CA* (1/8), *CTNNB1* (1/8), *PGR* (1/8), *ERBB4* (2/8), *ERBB3* (1/8), *BRCA1* (1/8)
Piscuoglio et al. (2015)[11],^a^	10	PCR amplification, Sanger sequencing	Triple-negative (10/10)	*TP53* (8/10), E542K *PIK3CA* (1/10)
Ripamonti et al. (2013) [27]	1	IHC, DNA sequencing	Triple-negative	EMA positive, GCDFP-15 positive *BRCA1* and *TP53* mutated
Reis-Filho et al. (2008) [46]	6	FISH	Triple-negative (6/6)	Negative for t(12; 15) (*ETV6⁄NTRK3* translocation)

α1-ACT – α1-antichymotrypsin; EMA – Epithelial membrane antigen; ER – Estrogen receptor; FISH – Fluorescent in situ hybridization; GCDFP-15 – Gross cystic disease fluid protein 15; HER2 – Human epidermal growth factor receptor; IHC – Immunohistochemistry; NGS - Next-generation sequencing; NTRK – Neurotrophic Tyrosine Receptor Kinase; PCR – Polymerase chain reaction; PR – Progesterone receptor; SNV – Single-nucleotide variant; WES – Whole-exome sequencing.

a The same samples were used.

Although reported in several studies [reviewed in Limite et al. [21]], the association between AcCC and microglandular adenosis as a precursor lesion is controversial [[45], [48], [49]]. For example, Rosen questions the existence of AcCC as a distinct entity and instead favors the entity “invasive carcinoma with acinic cell differentiation arising in microglandular adenosis” [48]. On the other hand, Geyer et al. have provided molecular evidence that microglandular adenosis and AcCC represent the low-grade spectrum of TNBC lesions with indolent clinical course and share some molecular features (e.g., *TP53* mutations and common copy number alterations, such as gains of 1q, 2q, 7p, and 8q and losses of 3p, 5q, 6q, 14q, 17p, and 17q, Table 3) [49,45]. Notably, both AcCC and microglandular adenosis share a similar immunophenotype, such as S-100 expression [4]. Occasionally, the difference between AcCC with predominant microglandular growth pattern and microglandular adenosis may also be challenging [42].

Other differential diagnoses of AcCC include invasive carcinomas NST, apocrine carcinomas, and oncocytic carcinomas [3,42]. Secretory carcinoma may be another mimicker, but it usually has bland nuclear morphology and harbors a balanced t(12; 15), causing *ETV6-NTRK3* fusion, which is absent in AcCC. Clues for correct diagnosis include cellular features and the presence of intracytoplasmic granules, as well as the expression of biomarkers of serous and acinar differentiation (e.g., lysozyme and α1-antichymotrypsin) [3,42] (Table 3). In apocrine carcinomas, the status of the androgen receptor (AR) and HER-2/neu, which are positive in all and amplified in 30–50%, respectively, is helpful [50,51]. Oncocytic carcinoma cells are strongly positive for mitochondrial antibodies and overexpress ER > 50% of cases [3].

### 1.4 Immunohistochemical profile of AcCC

The immunohistochemical characteristics of AcCC are summarized in Table 2, Table 3. AcCC is usually classified as triple-negative breast cancer, meaning that ER, PR, and Her-2/neu are negative (Fig. 2E). However, rare cases (∼10%) of ER and/or PR-positive AcCC have also been reported [7,41,10,31,[14], [22], [29], [34], [37]] (Table 3, Table 4). AR is also rarely positive (10%) [32].

Although Her-2/neu expression or HER-2/neu gene amplification is absent in AcCC, the epidermal growth factor receptor (EGFR or HER1) expression has been reported in one study [31]. Other members of the EGFR family (HER3 and HER4) have not been studied at the protein level, but their mutations have been reported (Table 3). Like other epithelial neoplasms, AcCC has positive staining for a broad spectrum of cytokeratins and low-molecular-weight cytokeratins (e.g., CK7 and CK18) (Table 3). Similar to TNBC NST with basal phenotype, high-molecular-weight cytokeratins (e.g., CK5/6) can also be detected in a subset of cases. S-100 and EMA positivity are also consistent features of AcCC, as are markers of serous and acinar differentiation (e.g., lysozyme, 1-anti-chymotrypsin/trypsin, and amylase) (Table 3).

The breast-specific markers GATA3 and GCDFP-15 were detected in ∼50% of AcCC (Fig. 2F). Despite the limited data, the loss of E-cadherin and β-catenin proteins has not yet been reported. However, two independent studies have detected mutations of the *CTNNB1* gene encoding β-catenin protein [19,44]. Mutations of the *CDH1* gene (encoding E-cadherin) have not been reported previously (Table 4). Interestingly, *CTNNB1* gene mutations have not been described in salivary AcCC but do occur in pancreatic acinar cell carcinomas [52]. TNBCs are also characterized by the activation of β-catenin protein (reduced membranous and increased nuclear expression) but without *CTNNB1* gene mutation [53].

The proliferation rate measured by Ki-67 shows a wide range of positivity (5–71%); however, most studies reported low to moderate Ki-67 expression (range 5–30%), consistent with mitotic activity on H&E slides and the intermediate clinical course of AcCC [3].

### 1.5 Molecular genomic characteristics of AcCC

The molecular genomic characteristics of AcCC in the breast are summarized in Table 4.

Because of its rarity, little information is available on the molecular genomic features of AcCC. Overall, no pathognomonic genomic alterations have been described in the AcCC of the breast. However, molecular studies have shown that mammary AcCC has a similar molecular profile to TNBC [20,45]; in contrast, molecular features typical of salivary gland AcCC (e.g., recurrent (t[4; 9][q13; q31]) genomic rearrangement) are generally absent in its mammary counterparts. Thus, *TP53* mutations appear to be the most consistent molecular event in AcCC; other genetic alterations (e.g., *PIK3CA* mutations) have also been described in a subset of cases [20,19,45]. *BRCA1* gene alterations (mutations and gene deletions) also characterize some breast AcCC [27,19,45,44]. Beca et al. performed whole-exome and RNA sequencing in three AcCC cases and reported mutations in genes related to homologous recombination and DNA repair in two AcCC cases and a pathogenic *MLH1* germline mutation in the third AcCC case [44]. Guerini-Rocco et al. (2015) studied eight cases (two pure AcCC and six mixed), used massively parallel sequencing (panel of 254 genes), and performed further validation by targeted amplicon and Sanger sequencing on microdissected samples. They found identical genomic alterations in AcCC-only and mixed cases (Table 4) [19]. Piscuoglio et al. [20] performed a comparative molecular study (breast carcinoma vs. salivary AcCC) with Sanger sequencing to investigate the frequency of *TP53* and *PIK3CA* mutations in two cohorts. Consistent with the TNBC profile, only mammary AcCC had *TP53* (80%) and *PIK3CA* (10%) mutations, whereas all twenty salivary AcCC had no somatic mutations in these two genes [20]. In another comparative study, Geyer et al. explored molecular genomic features of AcCC and microglandular adenosis of the breast. They reported similar genomic alterations in the two breast lesions (e.g., *TP53, BRCA1, PIK3CA*, and *INPP4B*) (Table 4) [45]. The repertoire of somatic mutations in both lesions was comparable to TNBC NST. Both lesions had multiple and complex gene copy changes, including gains of 1q, 2q, 7p, and 8q and losses of 3p, 5q, 6q, 14q, 17p, and 17q. However, loss of 16q in the entire arm was not found, consistent with high-grade TNBC NST. Amplifications within the 8q region were detected in both cohorts (affecting *FSBP, EPPK1, MYC, SLA*, and *COL14A1*) [45]. The authors concluded that both tumors represent a spectrum of “low-grade forms of TNBC with no/low metastatic potential”, although some have the potential to progress to high-grade forms of TNBC [45].

Recurrent genomic rearrangement [t(4; 9) (q13; q31)], which allows upregulation of the transcription factor Nuclear Receptor Subfamily 4 Group A Member 3 (*NR4A3*), has been described as an oncogenic driver event in salivary AcCC [54]. Several studies have shown that NR4A3 is an excellent diagnostic biomarker with high sensitivity and specificity in detecting salivary gland AcCC [[55], [56], [57], [58]]. In addition, Owosho et al. (2021) demonstrated a higher diagnostic utility of NR4A3 compared with DOG1 immunostaining for salivary gland AcCC [58]. No study has yet reported on the NR4A3 status for AcCC of the breast, though Waever et al. (2021) have reported DOG1 expression in one case of mammary AcCC [12]. The fact that t(12; 15) (*ETV6/NTRK3* translocation) typical of secretory carcinomas is not present in the AcCC of the breast [46] suggests that this finding might be useful for differential diagnosis in cases with overlapping or similar morphology.

### 1.6 Novel treatment options for AcCC patients

In addition to attempting neo-adjuvant and adjuvant chemotherapy (e.g. with adriamycin, cyclophosphamide, methotrexate and 5-fluorouracil (Table 2), the unique molecular and genomic profile of mammary AcCC might also allow for potential targeted therapies in these patients-though clinical information on this topic in the literature is limited. Similar to TNBC NST, *BRCA1* mutations have been reported in a subset of AcCC and mutations in genes related to homologous recombination and DNA repair and *MLH1* [44] (Table 4). Consequently, these patients may be viable candidates for treatment with poly(ADP-ribose) polymerase (PARP) inhibitors (PARPi) such as olaparib and talazoparib-both of which are already approved for metastatic breast cancer patients housing germline *BRCA1* mutations and HER2-negative tumors [59,60].

Dysregulation of the phosphoinositide 3 (PI3)-kinase/Akt signaling pathway including through activating *PIK3CA* mutations, that are among the most common genetic alterations in breast cancer, particularly in luminal breast tumors [61], is associated with increased cancer cell growth, proliferation and survival. The PIK3CA inhibitor alpelisib is a p110α-specific PI3K inhibitor that has a better safety profile than non-specific PI3K inhibitors. The U.S. Food and Drug Administration (FDA) has already approved its use in combination with fulvestrant for treating postmenopausal women with ER+/HER2-, *PIK3CA*-mutated breast cancer that has progressed after hormone therapy [62]. *PIK3CA* and/or *AKT1* mutations are also detectable in ∼25–30% of advanced TNBC NST and special types such as metaplastic breast carcinoma [63,64]. Recent clinical data also indicate a potential effect of targeted treatment for these breast cancer patients [65,66]. Given that a small proportion of mammary AcCC also has activating *PIK3CA* gene mutations, PIK3CA inhibitors should be considered as a treatment option for patients with advanced/or refractory disease.

The PI3K/Akt signaling pathway also acts as a downstream effector of the EGFR family of receptor tyrosine kinases in regulating cell growth, proliferation and survival [67]. The fact that some breast AcCC are reported to be EGFR positive (see Table 3), might imply that clinically-approved EGFR inhibitors, either as small molecule tyrosine kinase inhibitors like gefitinib or monoclonal antibodies like cetuximab, might also be indicated for these patients.

The immune checkpoint inhibitor pembrolizumab (Keytruda®, Merck) and predictive companion diagnostic (CDx) test (PD-L1 expression by immunohistochemistry, defined as Combined Positive Score [CPS] ≥10) have been approved for both high-risk early-stage TNBC and locally recurrent, unresectable/metastatic tumors [68]. In contrast to salivary glands AcCC [69], no study has reported PD-L1 receptor status in mammary AcCC. Similarly, the status of another predictable biomarker, tumor mutation burden (TMB) in breast AcCC remains unknown. Thus, in the absence of PD-L1 expression, the use of checkpoint inhibitors in mammary AcCC patients at this stage is not warranted.

In summary, several molecular targets observed in AcCC might lend themselves to potential targeted therapies with already clinically approved drugs that could also provide improved clinical outcomes in patients with this rare carcinoma.

## 2 Conclusions

Our comprehensive literature review confirms the rarity of mammary acinic cell carcinoma. Its clinical and radiologic features are similar to invasive ductal carcinoma NST, while its morphology may have various growth patterns. However, the cytologic features are specific because the cells contain zymogenic granules in the cytoplasm. Acinic cell carcinomas usually exhibit a triple-negative phenotype and less aggressive clinical behavior than the NST subtype. Molecular genomic features are similar to TNBC NST but are significantly different from AcCC arising in the salivary glands, which harbor a characteristic fusion. Based on the presence of *CTNNB1* mutations in mammary AcCC, this tumor may be closer to the pancreatic counterpart than to the salivary-type malignancy or TNBC. Biomarkers for targeted treatment are currently limited, although a few studies have identified potentially targetable biomarkers (e.g., *BRCA, PIK3CA*). These molecular targets may guide tailored therapeutics for individual cases with advanced/or refractory forms of acinic cell carcinoma.

## 3 Funding

The article-processing fee of this article was covered by the Qatar National Library (QNL).

## Declaration of competing interest

The authors declare no conflict of interest.
